# Complications of total laparoscopic hysterectomy: with vs. without a uterine manipulator

**DOI:** 10.1186/s12905-025-04093-4

**Published:** 2025-11-14

**Authors:** Erdener Karacan, Ilyas Turan, Özgür Ozan Ceylan, Mehmet Güney, Mehmet Okan Özkaya, Evrim Erdemoglu

**Affiliations:** 1 Department of Obstetrics and Gynecology, Isparta City Hospital, Isparta, Turkey; 2 Department of Gynecologic Oncology, Batman Training and Research Hospital Gültepe, Eflatun Cd. No:1, Batman, 72070 Turkey; 3 Department of Obstetrics and Gynecology, Turgutlu State Hospital, Manisa, Turkey; 4 Department of Obstetrics and Gynecology, Dokuz Eylül University Hospital, İzmir, Turkey; 5 Department of Obstetrics and Gynecology, Süleyman Demirel University Hospital, Isparta, Turkey; 6 Department of Gynecologic Oncology, Süleyman Demirel University Hospital, Isparta, Turkey

**Keywords:** Hysterectomy, Complications, Laparoscopy, Uterine manipulator, Laparotomy

## Abstract

**Background:**

Uterine manipulators may decrease the incidence of complications associated with total laparoscopic hysterectomy (TLH), but contrary views exist regarding the use of uterine manipulators for TLH. There are insufficient data on the safety of TLH without a uterine manipulator in both benign and malignant cases. Data on the safety and outcomes of TLH performed without a uterine manipulator remain limited. While some studies have investigated this issue, direct comparisons between TLH with and without the use of a manipulator, particularly across both benign and malignant indications, are still relatively scarce.

**Objective:**

The purpose of this study was to analyze the results and complications of TLH either with (group B) or without a uterine manipulator (group A), which was performed for benign and malignant indications.

**Study Design:**

A retrospective comparative study was conducted to evaluate the results of TLH with and without the use of a uterine manipulator. Group A included 138 patients, and Group B included 1300 patients. Patient characteristics, surgical indications, uterine weight, blood loss/transfusion, intraoperative urinary/intestinal complications, postoperative complications, and the rate of conversion to laparotomy were analyzed.

**Results:**

Patient characteristics and uterine weights were similar. There was a significant decrease in hemoglobin in group B (1.37 versus 1.52, *p* < 0.05). There was no significant difference in the transfusion rates between the two groups. The intraoperative complication rates were 4.3% in group A and 2.8% in group B (*p* > 0.05). After adjustment for malignant indication, group allocation was not significantly associated with intraoperative complications (OR = 0.57; 95% CI 0.23–1.42; *p* = 0.227). Rates of bladder, ureteral, and intestinal injuries were similar, although conversion to laparotomy was higher in group A (2.3% vs. 0%). Postoperative complication rates were also comparable between groups, and logistic regression showed no significant association after adjustment for malignancy (OR = 0.54; 95% CI 0.21–1.35; *p* = 0.184).

**Conclusion:**

Our findings indicate that TLH without a uterine manipulator can be safely performed in selected patients. However, as the procedure is technically more demanding and evidence remains limited, further multicenter prospective studies are needed to validate these results and guide appropriate patient selection.

**Trial registration:**

The study was conducted in accordance with the ethical standards set forth in the Declaration of Helsinki and approved by the Ethics Committee of Süleyman Demirel University (approval date: 08/06/2022, approval number: 12/165). Informed consent was obtained from the participants prior to participation.

## Introduction

Total laparoscopic hysterectomy (TLH) is a common operation for benign, urogynecological or malignant gynecologic diseases. Laparoscopy is associated with reduced postoperative pain, early ambulation and discharge, and minimal blood loss. This makes it an ideal procedure for those who desire to have surgery under a magnified view. After total laparoscopic hysterectomy, patients can be referred for adjuvant treatment without delay, depending on the stage of their malignancy. Moreover, in benign cases, TLH has been shown to improve patient comfort and significantly reduce hospital stay compared with abdominal hysterectomy [1].

The uterine manipulator assists the surgeon in performing a total laparoscopic hysterectomy by providing better vision and assisting with several steps of the operation. The use of a manipulator may protect surrounding structures, particularly the ureter [2]. Therefore, TLH is often performed with the help of a uterine manipulator. The uterine manipulator may facilitate certain steps in TLH by improving surgical exposure and tissue traction. However, its potential benefits in both benign and malignant cases remain a subject of debate. While shorter operative times and reduced blood loss have been reported in the literature, concerns persist in malignant cases regarding peritoneal tumor cell dissemination and iatrogenic pseudoinvasion. Moreover, outcomes may also be influenced by surgeon experience, case complexity, and perioperative management.

However, some centers avoid uterine manipulators in hysterectomies for benign and malignant diseases. Data on the safety and outcomes of TLH performed without a uterine manipulator remain limited. While some studies have investigated this issue, direct comparisons between TLH with and without the use of a manipulator, particularly across both benign and malignant indications, are still relatively scarce. In this study, we conducted a single-center retrospective comparison of TLH performed with and without the use of a manipulator, evaluating intraoperative and postoperative complication rates. The secondary outcomes included estimated blood loss, transfusion, conversion to laparotomy, and, in malignant cases, oncological safety parameters [3].

## Materials and methods

Data from 1438 patients who underwent total laparoscopic hysterectomy between January 15, 2017, and April 15, 2022, were retrieved. The division of gynecologic oncology performed TLH without a uterine manipulator, whereas the Department of Obstetrics and Gynecology performs TLH with a uterine manipulator. The study was approved by the institutional review board (08.06.2022, protocol number: 12/165). Patient characteristics, preoperative features, indications for operation, uterine weight, pre- and postoperative hemoglobin levels, blood transfusion, fresh frozen plasma transfusion, intraoperative urinary/intestinal complications, and postoperative complications were analyzed.

The study was conducted retrospectively in a referral center. Patient allocation to groups occurred naturally in clinical practice, as cancer patients were referred to the gynecologic oncology unit; therefore, no randomization was applied.

### Statistical analysis

All statistical analyses were performed using Jamovi (The jamovi project Version 2.6, https://www.jamovi.org). Descriptive statistics and figures were generated with the same software. Continuous variables are expressed as medians, and categorical variables as frequencies and percentages. Comparisons between groups were carried out using the chi-square test or Fisher’s exact test for categorical variables, and the Mann–Whitney U test for continuous variables, as no variable showed a normal distribution. Logistic regression analysis was additionally performed to adjust for potential confounding, particularly malignant indications, and to evaluate the association between clinical variables and outcomes. Due to the retrospective design of the study and the fixed sample sizes in both groups, no a priori power analysis was conducted; instead, comparisons were evaluated on the basis of confidence intervals, and a p value < 0.05 was considered statistically significant.

### Surgical technique

The patient is placed in the low lithotomy position with the left arm tucked alongside the body. After the establishment of a pressure pneumoperitoneum of 15 mmHg with a Veress needle, a 10 mm trocar is inserted into the umbilicus for the optic. The table is positioned in the Trendelenburg position at an angle of 20–35 degrees. Two lateral ancillary 5 mm trocars are inserted in the lower left and right quadrants. In addition, an additional 5 mm suprapubic trocar is inserted. An additional 5–10 mm trocar was positioned at the left palmar point in patients who underwent lymphadenectomy and other upper abdominal surgeries. The intra-abdominal pressure was set to 11–12 mmHg after the insertion of the trocars.

#### TLH without a uterine manipulator - group A

The retroperitoneum was opened parallel to the infundibulopelvic ligament on both sides. The external iliac artery and vein, ureter, internal iliac artery, and umbilical artery were identified. The pararectal and paravesical fossa is developed, and the uterine artery is isolated for at least a few centimeters. Then, the uterine artery is coagulated and transected at its origin from the internal iliac artery. The ureter is released from the posterior leaf of the broad ligament. A window is formed between the ovarian vessels and the ureter. The infundibulopelvic ligament is coagulated and cut. A space was then created between the ureter and the sacrouterine ligaments, and the dissection was carried out in the rectovaginal space. Round ligaments are coagulated and transected, and the bladder flap is developed. After entering the vesicovaginal space, the blade of the Breisky retractor or an ovarian forceps holding two gauze sponges helps to dissect the plane and determine the colpotomy site. The uterine vessels and cardinal ligament were coagulated and cut below the level of the colpotomy site. A harmonic scalpel or monopolar cautery is used to make an incision at the level of the Breisky retractor in the vagina. During colpotomy, the uterus is manipulated with forceps in the ancillary trocars. In the case of a large uterus, vaginal morcellation may be performed with scissors. The vaginal cuff is closed with 0 Vicryl, and a drain is left if it is indicated.

There are several basic instruments for surgery: a monopolar hook, a bipolar overhold, scissors, a Breisky retractor, and one traumatic, traumatic grasper. If advanced bipolar instruments (LigaSure™ 5-mm Blunt Tip Laparoscopic Instrument, Medtronic, Dublin, Ireland) or ultrasonic scalpels (Sonicision™ Curved Jaw Cordless Ultrasonic Dissection System, Medtronic, Dublin, Ireland) were available at the time of surgery, they were used.

##### TLH with a uterine manipulator - group B

A uterine manipulator is used to expose anatomical structures and the pelvis. After visualization of the ureter under the peritoneum, the round ligament and ovarian vessels close to the ovary are coagulated and transected. The vesicouterine space is entered and developed while the uterine manipulator pushes the uterus cephalad. The ascending branch of the uterine artery on the uterine corpus is coagulated and transected via ligatures. The uterine vessel and cardinal ligaments are coagulated and transected while the uterine manipulator pushes the uterus cephalad. This maneuver helps to grasp the pedicles. Vaginal colpotomy is performed with the aid of a uterine manipulator. An ultrasonic scalpel was used to incise the vagina. The vaginal cuff is sutured with 0 vicryl, and a drain is left if it is indicated.

The instruments used included an advanced bipolar instrument, an ultrasonic scalpel, a bipolar overhold, scissors, a Breisky retractor, and one traumatic grasper; traumatic grasper are the basic instruments used. For uterine manipulation, either Clermont Ferrand (Karl Storz Gmbh & Co., Tuttlingen, Germany) or RUMI (Cooper Surgical, Shelton, Connecticut) was used. When they were not available, a uterine curette was used to manipulate the uterus.

## Results

A total of 138 TLHs were carried out without the use of a manipulator, and 1300 TLHs were carried out using a uterine manipulator. The median age of patients in group A was 52 (range: 32–84) years, and that in group B was 51 (range: 33–84) years (*p* > 0.05). The indications for TLH differed between the two groups. A total of 18.3% of patients in group A underwent surgery for malignancy, whereas only 2% of women in group B underwent surgery for cancer (*p* < 0.001). Previous conization and TLH for cervical preinvasive lesions were more common in Group A (13.8%) than in Group B (6.2%) (*p* < 0.001). The TLH indications for benign gynecological diseases also differed between the two groups (Table 1 ). The rates of additional procedures were similar in the two groups, but the types of additional surgeries differed (Table 2 ).

**Table 1:** Indications for total laparoscopic hysterectomy* (*Chi-square test was used; fisher’s exact test was applied when expected cell counts were < 5.)

	Group A n:138	Group B n:1300	*p* value
Cancer	%18.8(26)	%2(26)	*P* < 0,001
Uterin fibroids	%20.3(28)	%45.7(594)	*P* < 0,001
Pelvic organ prolapsus	%3.6(5)	%20.1(261)	*P* < 0,001
EIN/endometrial hyperplasia	%32.6(45)	%25.8(336)	*p* > 0,05
Cervical preinvasive disease	%13.8(19)	%6.2(80)	*P* < 0,001
Adnexal mass	%8(11)	%9.1(118)	*P* < 0,05
Abnormal uterine bleeding	%21.7(30)	%31.5(410)	*P* < 0,05
Chronic pelvic pain	%1.4(2)	%1.8(24)	*P* > 0,05
Endometriosis	%0.7(1)	%2.8(36)	*p* > 0,05

**Table 2:** Additional surgical procedures performed in conjunction with total laparoscopic hysterectomy (POP: pelvic organ prolapse) (Chi-square test was used; fisher’s exact test was applied when expected cell counts were < 5.)

	Group A n:138	Group B n:1300	*p* value
Additional procedures	%15,9(22)	%23,5(305)	*p* > 0,05
Urogynecologic surgery	%0,7(1)	%15,2(197)	*p* < 0,001
POP surgery	%2,9(4)	%15,5(201)	*p* < 0,001
Lymphadenectomy	%15,2(21)	%0,2(2)	*p* < 0,001

**Fig. 1:**
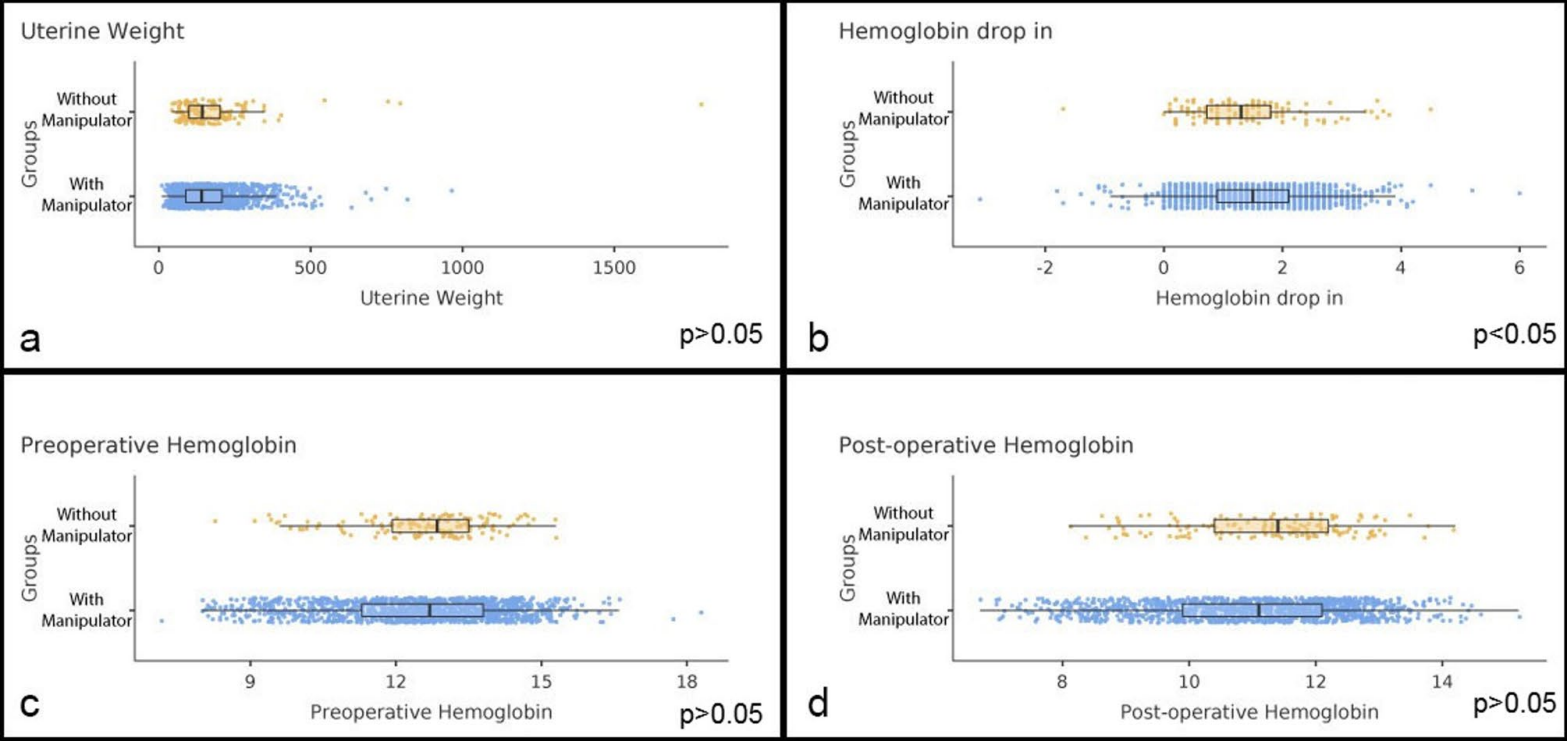
Uterine weight and preoperative and postoperative hemoglobin levels and hemoglobin drop-in values between the groups. **a** Uterine weight. **b** Hemoglobin drop-in value. **c** Preoperative hemoglobin value. **d** Post-operative hemoglobin values (Mann–Whitney U test was used)

**Fig. 2:**
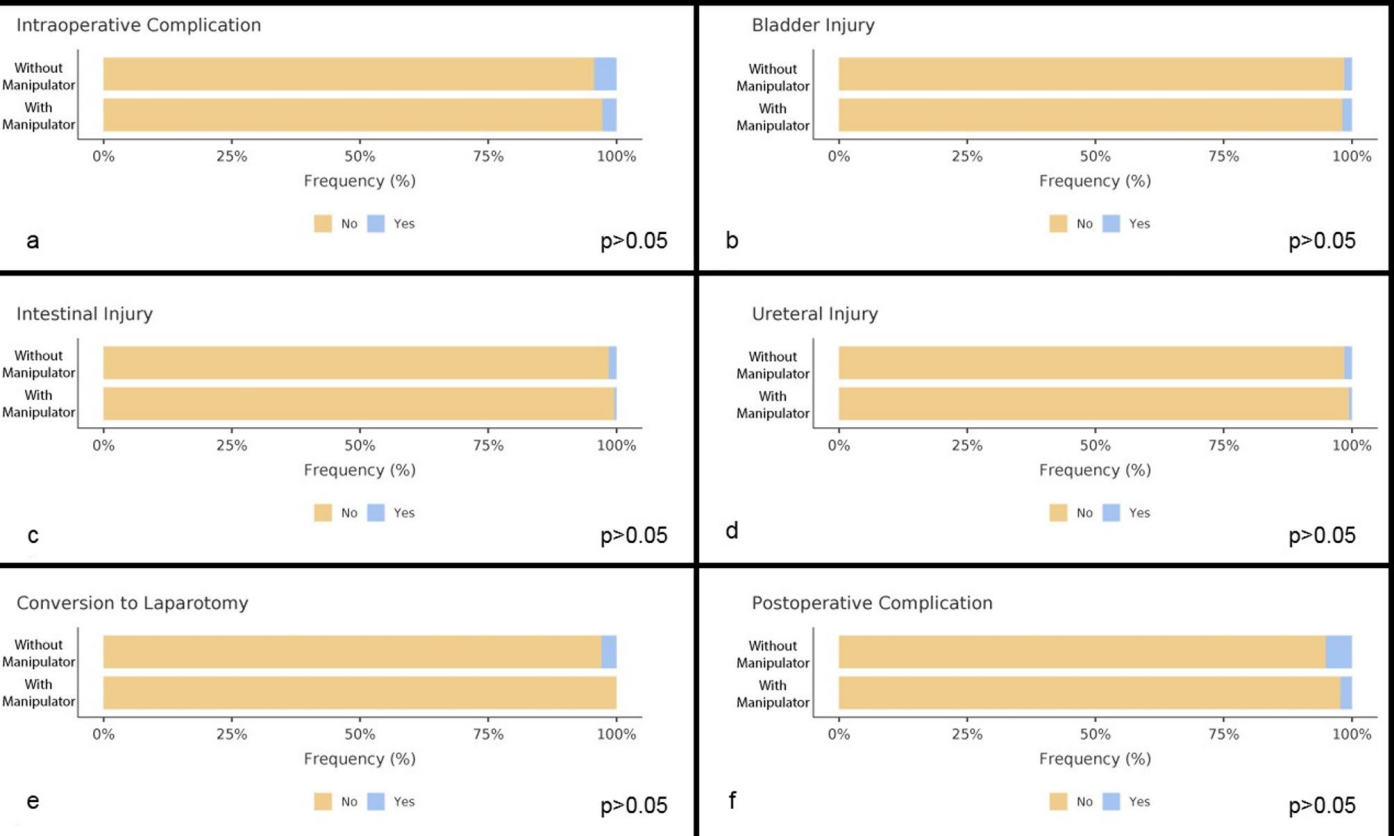
Complication rates between groups. **a** Frequency of intraoperative complications. **b** Frequency of intraoperative bladder injury. **c** frequency of intraoperative intestinal injury. **d** Frequency of intraoperative ureteral injury. **e** frequency of conversion to laparotomy. **f** Frequency of postoperative complications. (Chi-square test was used; Fisher’s exact test was applied when expected cell counts were < 5.)

The uterine weight, preoperative hemoglobin level, and postoperative hemoglobin level were similar (*p* > 0.05); however, there was a significant decrease in hemoglobin in group B (1.37 versus 1.52, *p* < 0.05). Although the difference in postoperative hemoglobin drop between the groups reached statistical significance, the absolute difference of 0.15 g/dL is unlikely to be of clinical relevance. Such a minor reduction does not influence transfusion requirements, postoperative morbidity, or patient management. Therefore, this finding should be interpreted as a statistical observation rather than a clinically meaningful outcome. Figure 1 There was no significant difference in the transfusion rates, median number of erythrocyte transfusions, or median number of fresh frozen plasma transfusions between the two groups. (Table 3) The intraoperative complication rates were 4.3% in group A and 2.8% in group B (*p* > 0.05). Intraoperative complications were defined to include bladder, ureteral, and intestinal injuries; thus, the overall intraoperative complication rate reflects the occurrence of any one or more of these events. In a bivariable logistic regression, after adjusting for malignant indications, the group (B vs. A) was not significantly associated with intraoperative complications (OR = 0.57; 95% CI 0.23–1.42; *p* = 0.227). Malignancy was also not associated with intraoperative complications (OR = 0.50; 95% CI 0.06–3.88; *p* = 0.504). The rates of bladder, ureteral, and intestinal injuries were similar between the two groups, but the rate of conversion to laparotomy was higher in group A (2.3%) than in group B (0%). The higher rate of conversion to laparotomy in group A may be attributed to the technical challenges of performing TLH without a manipulator. In our series, concomitant procedures were included in the same surgical session, which may also have contributed to the increased conversion rate. Figure 2. Postoperative complication rates were similar in groups A and B. According to the bivariable logistic regression, after adjustment for malignant indications, the group (B vs. A) was not significantly associated with postoperative complications (OR = 0.54; 95% CI 0.21–1.35; *p* = 0.184). Malignancy was associated with higher odds but did not reach statistical significance (OR = 2.65; 95% CI 0.82–8.56; *p* = 0.104). Since the proportion of malignant indications differed between the groups, malignancy was included in the model as a potential confounder.

**Table 3:** Comparison of surgical outcomes and complication rates between group A and group B (Values are presented as mean ± standard deviation (minimum–maximum) or percentage (number of cases).) (Chi-square test was used for categorical variables fisher’s exact test when expected cell counts were < 5, Mann–Whitney U test was used for continuous variables, depending on data distribution.)

	Group A n:138	Group B n:1300	*p* value
Uterine weight (g), mean ± SD (min–max)	181 ± 176 (43.5–1787)	163 ± 102 (10.2–965)	*p* > 0.05
Preoperative hemoglobin (g/dL), mean ± SD (min–max)	12.6 ± 1.35 (8.3–15.3)	12.5 ± 1.75 (7.2–18.3)	*p* > 0.05
Postoperative hemoglobin (g/dL), mean ± SD (min–max)	11.2 ± 1.30 (8.1–14.2)	11.0 ± 1.52 (6.7–15.2)	*p* > 0.05
Hemoglobin difference (g/dL), mean ± SD (min–max)	1.37 ± 0.92 (–1.7–4.5)	1.52 ± 0.91 (–3.1–6.0)	*p* < 0.05
Intraoperative complication	%4,3(6)	%2,8(36)	*p* > 0.05
Postoperative complication	%5,1(7)	%2,2(29)	*p* > 0.05
Blader injury	%1,4(2)	%1,8(24)	*p* > 0.05
Intestinal injury	%1,4(2)	%0,5(6)	*p* > 0.05
Ureteral injury	%1,4(2)	%0,5(7)	*p* > 0.05
Laparotomy	%2,9(4)	%0(0)	*p* < 0.001

## Discussion

### Principal findings

Our study demonstrated that total laparoscopic hysterectomy (TLH) without a uterine manipulator can be safely performed for both benign and malignant indications. The overall complication profile, including urinary tract and visceral injuries, was comparable to that of procedures performed with a manipulator. These results support the concept that surgical safety primarily depends on adequate exposure and mastery of retroperitoneal anatomy rather than on the use of a manipulator itself. In our technique, the retroperitoneum is opened and extensive ureterolysis is performed along the posterior leaf of the broad ligament, followed by uterine artery transection at its origin—steps that allow clear visualization and secure control of vital structures. Although this approach requires a thorough understanding of pelvic anatomy and may be associated with a steeper learning curve, it offers a standardized and reproducible method that ensures safety without compromising surgical outcomes.

The learning curve for TLH performed without a uterine manipulator is likely to be longer, especially for surgeons in training or those less experienced in retroperitoneal dissection. However, once the anatomical landmarks and dissection planes are well understood, the procedure becomes straightforward and efficient. Therefore, structured training programs emphasizing retroperitoneal anatomy, vascular identification, and stepwise dissection techniques are essential to achieve proficiency and minimize complications during TLH performed without a uterine manipulator.

### Results in the context of what is known

TLH without a uterine manipulator can also be safely performed in patients with a uterine mass greater than 250 g. Mebes et al. reported their experience in treating 52 women with a uterus > 250 g operated without a uterine manipulator at the University of Schleswig-Holstein; they concluded that complication rates were comparable, blood loss was minimal, no conversions to laparotomy occurred, and concomitant procedures were postponed to secondary operations to maintain surgical standardization [4]. Our study revealed that TLH without a uterine manipulator did not increase intraoperative bladder, ureteral, or intestinal injury rates or postoperative complications. In our patients, concomitant procedures (such as lymphadenectomy) were completed during the same operation, and no secondary complementary surgery was planned, which may have contributed to the higher rate of conversion to laparotomy. Kavallaris et al. reported their technique of TLH without the use of a uterine manipulator in 67 patients. This technique is similar to the technique we used in our study [5]. Instead of a vaginal tube, they use their fingers to delineate the cervix. They reported that TLH without the use of any uterine manipulators seems to be a safe and practical surgical option, especially for patients with vaginal stenosis or who are nulliparous and for patients with an enlarged uterus. Our findings revealed that TLH without a uterine manipulator is safe and practical.

### Research and clinical implications

There is an ongoing debate about the use of uterine manipulators in the treatment of cervical cancer and endometrial cancer. The main disadvantage of the uterine manipulator in endometrial cancer is that it can cause positive peritoneal cytology and iatrogenic lymphovascular invasion and interfere with the pathologic assessment of the sample [6–8]. In a more recent nationwide population-based retrospective study conducted by Ye et al., it was demonstrated that the use of an intrauterine manipulator or performing surgery without a manipulator did not affect disease-free survival or overall survival in patients with low-grade endometrial cancer[9]. Following the results of the LACC trial, which reported that minimally invasive surgery decreases survival for cervical cancer patients, the Succor study was conducted to analyze the effect of the uterine manipulator on cervical cancer survival [10, 11]. In this trial, avoiding tumor spread at the time of colpotomy and avoiding the uterine manipulator in minimally invasive surgery were both associated with outcomes similar to those of open surgery. The focus of our study was not the effects of uterine manipulators on survival. Our findings show that TLH can be performed without a uterine manipulator in malignant cases. However, current evidence on whether the use of uterine manipulators worsens oncological outcomes remains inconclusive in the literature. A recent systematic review and meta-analysis by Scutiero et al. reported that, in minimally invasive surgery for endometrial cancer, the use of a uterine manipulator was not associated with increased risks of lymphovascular space invasion (LVSI) or recurrence. In addition, no significant difference was observed in the incidence of positive peritoneal cytology between manipulated and nonmanipulated cases [12]. In the study by Gendia et al., manipulator-free hysterectomy was performed not only in oncologic cases but also in benign cases, and the outcomes were comparable to those observed in our study [13].

Uterine artery closure at the uterus and at the internal iliac artery was compared in a randomized trial by Ucella et al. They concluded that occlusion of the uterine artery at the origin reduces intraoperative blood loss during TLH and appears to be more reproducible than occlusion at the uterus without higher complication rates [14]. In group A, intraoperative blood loss was lower than that in group B, potentially owing to occlusion and division of the uterine arteries at their origin from the internal iliac artery. While the intergroup difference in postoperative hemoglobin levels may be attributable to this surgical technique, the absolute difference was small (0.15 g/dL) and is unlikely to be clinically meaningful—e.g., it would not be expected to alter transfusion requirements or postoperative management.

Our findings may support the broader adoption of TLH without a manipulator in centers with advanced laparoscopic expertise, particularly in patients with distorted pelvic anatomy or when the use of a manipulator is contraindicated.

### Strengths and limitations

Some limitations and drawbacks exist with our study. This was a retrospective study without random selection. As cancer patients were referred to the gynecologic oncology unit of the referral center, randomization was not possible. We acknowledge this as a limitation of the study and emphasize that further randomized controlled trials are needed to confirm and strengthen our findings. However, all of the surgeries were performed in a standardized manner. There were other surgical procedures in 16.7% of women in group A and 23.5% of women in group B. This was associated with different indications of TLH in the groups. Therefore, it might be difficult to extract the results of the study only about TLH. However, our study with this design may reflect a more realistic conclusion since TLH is usually performed in addition to other gynecologic procedures. In addition, a higher rate of gynecological oncological procedures is generally expected to be associated with more complications, but the rates of complications, blood transfusions, and blood loss were similar for TLH without a uterine manipulator, even though the indications for malignancy were greater in this study group. The retrospective design of our study limited our ability to obtain certain information. We did not perform a comparison of operative times between the two groups because the recorded duration represented the entire surgical procedure, including additional gynecologic interventions performed at the same session. As these concomitant procedures vary considerably among patients, the recorded times do not reflect the isolated duration of TLH itself, which would have limited the validity and consistency of any comparison. Isolated perforations caused by the manipulator, without injury to other organs, were generally not reported in the operative notes because they were not regarded as clinically significant. Although complications should ideally be defined according to a standardized classification system, the retrospective design of the study may have limited the accuracy of patient records and the categorization of complications. In addition, the lack of data on long-term complications represents an important limitation of our study.

## Conclusion

Our findings suggest that total laparoscopic hysterectomy (TLH) without a uterine manipulator can be performed safely in selected patients. However, broader conclusions should be made with caution until these results are confirmed by multicenter prospective studies. In this study, we aimed to show that TLH can be safely performed without a uterine manipulator, regardless of its advantages or disadvantages. Although we did not focus on this issue directly, performing TLH without a manipulator is technically more demanding and requires a longer learning curve. Therefore, structured training and close supervision are essential, especially for less experienced surgeons.

Removing the uterine manipulator may have some benefits, such as reducing the risk of uterine perforation, preventing tumor spillage in oncologic cases, and simplifying the setup in patients with difficult pelvic anatomy. On the other hand, limited uterine mobility can make the dissection and exposure of surgical planes more challenging.

Further multicenter and prospective studies with larger samples are needed to confirm our results and to better define patient selection, surgical outcomes, and training methods. Until stronger evidence becomes available, the decision to perform TLH without a uterine manipulator should be made on a case-by-case basis, considering the surgeon’s experience and the patient’s clinical condition.

## Data Availability

The datasets used and/or analyzed during the current study are available from the corresponding author on reasonable request.
